# *ABHD5* frameshift deletion in Golden Retrievers with ichthyosis

**DOI:** 10.1093/g3journal/jkab397

**Published:** 2021-11-15

**Authors:** Sarah Kiener, Dominique J Wiener, Kaitlin Hopke, Alison B Diesel, Vidhya Jagannathan, Elizabeth A Mauldin, Margret L Casal, Tosso Leeb

**Affiliations:** Institute of Genetics, Vetsuisse Faculty, University of Bern, 3001 Bern, Switzerland; Dermfocus, University of Bern, Bern 3001, Switzerland; Department of Veterinary Pathobiology, Texas A&M University, College of Veterinary Medicine and Biomedical Sciences, College Station, TX 77843-4467, USA; Department of Small Animal Clinical Sciences, Texas A&M University, College of Veterinary Medicine and Biomedical Sciences, College Station, TX 77843-4474, USA; Department of Small Animal Clinical Sciences, Texas A&M University, College of Veterinary Medicine and Biomedical Sciences, College Station, TX 77843-4474, USA; Institute of Genetics, Vetsuisse Faculty, University of Bern, 3001 Bern, Switzerland; Department of Clinical Sciences and Advanced Medicine, School of Veterinary Medicine, University of Pennsylvania, Philadelphia, PA 19104, USA; Department of Clinical Sciences and Advanced Medicine, School of Veterinary Medicine, University of Pennsylvania, Philadelphia, PA 19104, USA; Institute of Genetics, Vetsuisse Faculty, University of Bern, 3001 Bern, Switzerland; Dermfocus, University of Bern, Bern 3001, Switzerland

**Keywords:** *Canis lupus familiaris*, dog, dermatology, genodermatosis, metabolism, lipid storage disorder, animal model, precision medicine, veterinary medicine

## Abstract

Ichthyoses are hereditary skin disorders characterized by the formation of scales and defects in the outermost layer of the epidermis. In dogs, at least six different breed-specific ichthyoses including a relatively common *PNPLA1*-related autosomal recessive ichthyosis in Golden Retrievers are known. In this study, we investigated 14 Golden Retrievers with scales that were not homozygous for the mutant *PNPLA1* allele suggesting a genetically distinct new form of ichthyosis. Histopathological examinations showed lamellar, orthokeratotic hyperkeratosis, and mildly hyperplastic epidermis that led to the diagnosis of a nonepidermolytic ichthyosis. Combined linkage and homozygosity mapping in 14 cases and 30 nonaffected family members delimited a critical interval of ∼12.7 Mb on chromosome 23. Whole-genome sequencing of an affected dog revealed a single protein-changing variant within this region that was not present in 795 control genomes. The identified variant is a 14 bp deletion in the *ABHD5* gene (c.1006_1019del), leading to a frameshift and altering the last 14 codons p.(Asp336Serfs*6). The genotypes at this variant showed perfect cosegregation with the ichthyosis phenotype in a large family comprising 14 cases and 72 controls. *ABHD5* encodes an acyltransferase required for lipid metabolism. In humans, variants in *ABHD5* cause Chanarin-Dorfman syndrome, a neutral lipid storage disease with ichthyosis. Our data in dogs together with the knowledge on the effects of *ABHD5* variants in humans strongly suggest *ABHD5*:c.1006_1019del as candidate causative genetic variant for a new canine form of ichthyosis, which we propose to designate as Golden Retriever ichthyosis type 2 (ICH2).

## Introduction

Ichthyoses comprise a heterogeneous group of cornification disorders characterized by generalized dry and scaly skin. Various genetic defects have been described, all disrupting the skin barrier and leading to hyperkeratosis and scaling of the skin (Oji *et al.* 2010; Schmuth *et al.* 2013). The classification of ichthyoses distinguishes between syndromic and nonsyndromic forms. Nonsyndromic ichthyoses refer to those with the phenotypic manifestation of the disease limited to the skin whereas syndromic ichthyoses additionally involve other organs. Ichthyoses can be further subdivided into epidermolytic and nonepidermolytic ichthyoses. This differentiation is light microscopy based. Epidermolytic ichthyoses are accompanied by epidermolytic hyperkeratosis at the ultrastructural level. Typical findings are intracellular vacuolization and formation of small intraepidermal blisters (Oji *et al.* 2010).

In humans, at least 67 different genes have been described to be related with different forms of ichthyosis (Uitto *et al.* 2020). In dogs, several breed-specific ichthyoses have been described. However, in only six of the canine ichthyoses, the underlying genetic defect has been identified (Mauldin 2013; Leeb *et al.* 2017). A heterozygous missense variant in *ASPRV1* caused an autosomal dominant form of nonepidermolytic ichthyosis in a German Shepherd (Bauer *et al.* 2017; OMIA 002099-9615). A mild epidermolytic ichthyosis in Norfolk terriers is caused by a splice-site variant in *KRT10* (Credille *et al.* 2005; OMIA 001415-9615). In American Bulldogs, a frameshift deletion in *NIPAL4* was found to cause autosomal recessive congenital ichthyosis (Casal *et al.* 2017; OMIA 001980-9615). A splice site variant in *SLC27A4* was described in Great Danes with a clinically severe, autosomal recessive syndromic ichthyosis (Metzger *et al.* 2015; OMIA 001973-9615). An autosomal recessive nonepidermolytic ichthyosis in Jack Russell Terriers is caused by a LINE-1 insertion into the *TGM1* gene (Credille *et al.* 2009; OMIA 000546-9615). Probably the most common canine ichthyosis is an autosomal recessive ichthyosis in Golden Retrievers (OMIA 001588-9615). The first case report is from 2004 (Hall and Yager 2004) and the disease phenotype has been well characterized in the following years (Guaguère *et al.* 2007, 2009; Cadiergues *et al.* 2008; Mauldin *et al.* 2008). The underlying genetic defect is a homozygous insertion-deletion (indel) variant in *PNPLA1* encoding patatin like phosphatase domain containing 1. The protein plays a key role in lipid organization and metabolism of the epidermal barrier and the defective protein in the affected Golden Retrievers causes malformation of the intercellular stratum corneum lipid layer and abnormal desquamation (Grall *et al.* 2012).

In this study, we investigated 14 Golden Retrievers with clinical and histopathological signs of nonepidermolytic ichthyosis. Despite the phenotypic similarity to the *PNPLA1*-related ichthyosis, none of these dogs carried the mutant *PNPLA1* allele in a homozygous state. In the present study, we therefore aimed to characterize this presumably new form of inherited ichthyosis and to unravel the causative genetic variant. We propose to term this specific phenotype Golden Retriever ichthyosis type 2 (ICH2, OMIA 002368-9615).

## Materials and methods

### Ethics statement

The Golden Retrievers in this study were privately owned and skin biopsies and blood samples for diagnostic purposes were collected with the consent of their owners. The collection of blood samples was approved by the *Cantonal Committee for Animal Experiments* (Canton of Bern; permit BE71/19). All animal experiments were done in accordance with local laws and regulations.

### Clinical and histopathological examinations

A physical examination was performed by the attending veterinarians. Two to four 4–6 mm skin punch biopsies per dog were taken and routinely processed for histopathology. Hematoxylin and eosin (H&E) stained slides were reviewed by board certified veterinary pathologists (DJW and EAM).

### Animal selection for genetic analyses

This study included 482 Golden Retrievers. They comprised 86 closely related dogs including 14 ICH2 affected and 72 unaffected relatives originating from North America. The remaining 396 dogs were Golden Retrievers of European origin from the Vetsuisse Biobank.

### DNA extraction

Genomic DNA was extracted from EDTA blood using the Maxwell^®^ RSC Whole Blood DNA with the Maxwell^®^ RSC instrument (Promega, Dübendorf, Switzerland).

### Linkage analysis and homozygosity mapping

Genotype data for 44 Golden Retrievers comprising dogs from 7 litters and their parents were obtained with 220 k Illumina CanineHD BeadChips by Geneseek/Neogen (Supplementary File S1, 11 unaffected parents, 14 affected, and 19 unaffected offspring). For all dogs, the call rate was >95%. Using PLINK v1.9 (Purcell *et al.* 2007), markers on the sex chromosomes or with unknown positions were removed. We further removed markers that had missing genotypes in any of the 44 dogs, Mendel errors, or a minor allele frequency <0.01. The final pruned dataset contained 110,720 markers and was organized into four separate subfamilies comprising between 5 and 23 dogs. To analyze the data for parametric linkage, an autosomal recessive inheritance model with full penetrance, a disease allele frequency of 0.55 and the Merlin software (Abecasis *et al.* 2002) were applied.

For homozygosity mapping, the genotype data for the 14 ICH2 affected dogs were used. Markers with call rates <100% and markers on the sex chromosomes were excluded. The --homozyg-group option in PLINK was used on a final dataset of 198,921 markers to search for extended regions of homozygosity. The output intervals were matched against the intervals from linkage analysis in Excel spreadsheets to find overlapping regions (Supplementary Table S1). A tped-file containing the markers on chromosome 23 was visually inspected in an Excel spreadsheet to precisely delimit the homozygous shared haplotype in the cases (Supplementary Table S1). All positions correspond to the CanFam3.1 reference genome assembly.

### Whole-genome sequencing

An Illumina TruSeq PCR-free library with an insert size of ∼330 bp was prepared from one ICH2 affected dog and sequenced at 26x coverage on an Illumina NovaSeq 6000 instrument. The reads were mapped to the dog CanFam3.1 reference genome assembly as previously described (Jagannathan *et al.* 2019). The sequence data were deposited under study accession PRJEB16012 and sample accession SAMEA8797074 at the European Nucleotide Archive.

### Variant calling and variant filtering

Variant calling was performed using GATK HaplotypeCaller (McKenna *et al.* 2010) in gVCF mode as described (Jagannathan *et al.* 2019). To predict the functional effects of the called variants, the SnpEff software (Cingolani *et al.* 2012), together with NCBI annotation release 105 for the CanFam 3.1 genome reference assembly was used. For filtering of private variants, we used 795 control genomes (Supplementary Table S2). Numbering within the canine *ABHD5* gene corresponds to the NCBI RefSeq accession numbers XM_542689.5 (mRNA) and XP_542689.2 (protein).

### Targeted genotyping

We used Sanger sequencing to confirm the candidate variant *ABHD5*:c.1006_1019del. A 389 bp (or 375 bp in case of the mutant allele) PCR product was amplified from genomic DNA using AmpliTaqGold360Mastermix (Thermo Fisher Scientific, Waltham, MA, USA) and primers 5′-CTG CTG GCC CTG TCA TTA GT-3′ (Primer F) and 5′-CAG GCT CTC TCT CCC ACA TT-3′ (Primer R). After treatment with exonuclease I and alkaline phosphatase, we sequenced the amplicons in both directions on an ABI 3730 DNA Analyzer (Thermo Fisher Scientific Corporation, Waltham, MA, USA). Sanger sequences were analyzed with the Sequencher 5.1 software (Gene Codes Corporation, Ann Arbor, MI, USA). Subsequently, targeted genotyping of dogs was performed by fragment length analysis of PCR products on a 5200 Fragment Analyzer (Agilent, Basel, Switzerland).

The *PNPLA1*:c.1445_1447delinsTACTACTA variant causing another form of ichthyosis in Golden Retrievers (Grall *et al.* 2012) was genotyped by Sanger sequencing of PCR amplicons as described above. A 300 bp PCR product (or 305 bp in case of the mutant allele) was amplified with the primers 5’-GGC CCT GAT AGT GAA GGA CA-3’ (Primer F) and 5’-TCC TAA CAC CTG CTC CTG CT-3’ (Primer R). The reverse primer was used for sequencing.

## Results

### Clinical description

**Figure 1:**
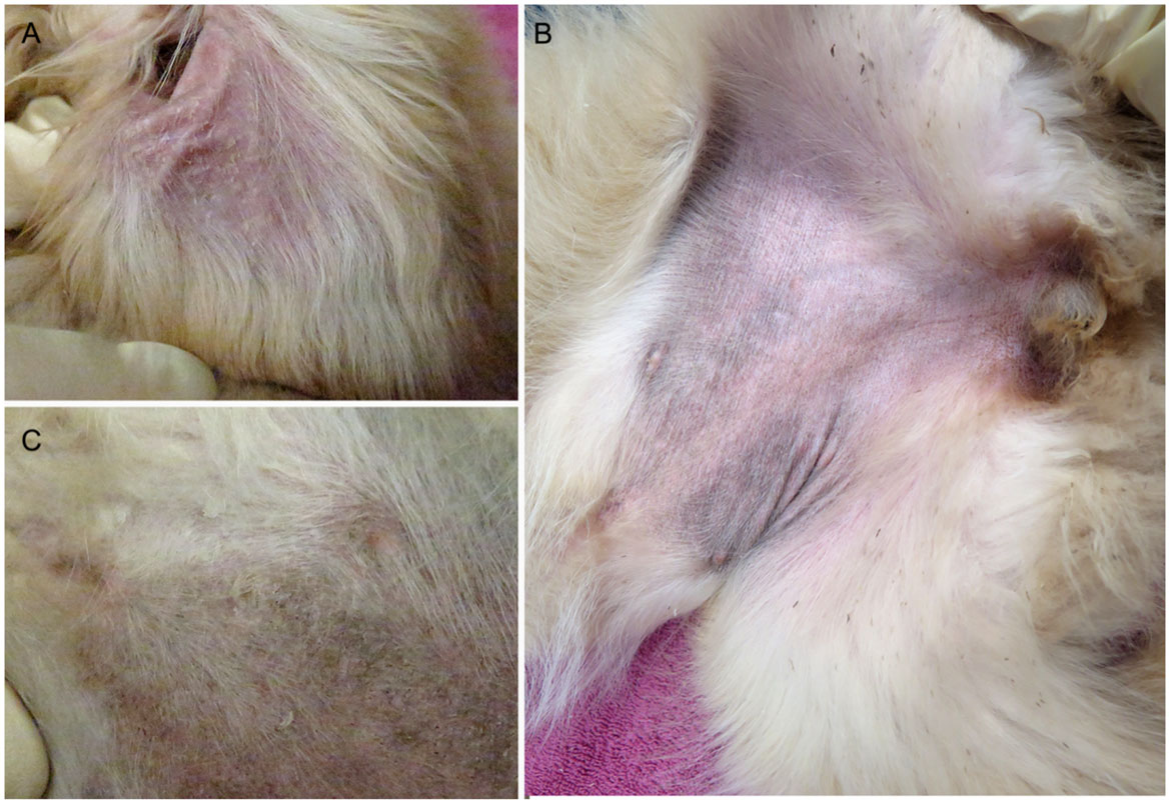
Clinical images from an 11-week-old Golden Retriever with ICH2. (A) Adherent scales on the inner pinna. (B) Thick scales on the ventral thorax and (C) abdominal hyperpigmentation.

ICH2 affected dogs had large white to gray and powdery to adherent scale throughout the hair coat. The abdominal skin was mildly hyperpigmented. Thick white scale was adherent to the concave surface of the pinnae (Figure 1).

### Histopathological examination

**Figure 2:**
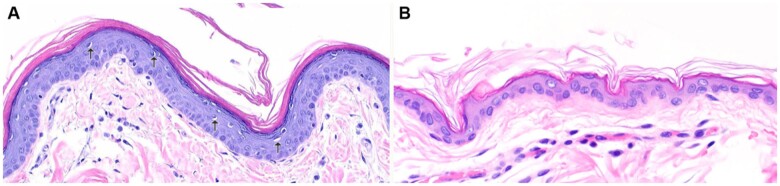
Histopathology of a Golden Retriever with ICH2. (A) Skin biopsy from an affected dog reveals marked thickening of the epidermis with expansion of the stratum corneum by laminar orthokeratotic hyperkeratosis. Numerous keratinocytes have perinuclear clear spaces (arrows). (B) Normal skin from an unaffected Golden Retriever. H&E 200X.

The skin biopsies of affected dogs showed moderate epidermal hyperplasia without dermal inflammation. The main change was expansion of the stratum corneum by laminar orthokeratotic hyperkeratosis. Numerous keratinocytes in the granular layer contained perinuclear clear spaces (Figure 2). The clinical signs together with the histopathological findings led to the diagnosis of a nonepidermolytic ichthyosis.

### Genetic analysis

**Figure 3:**
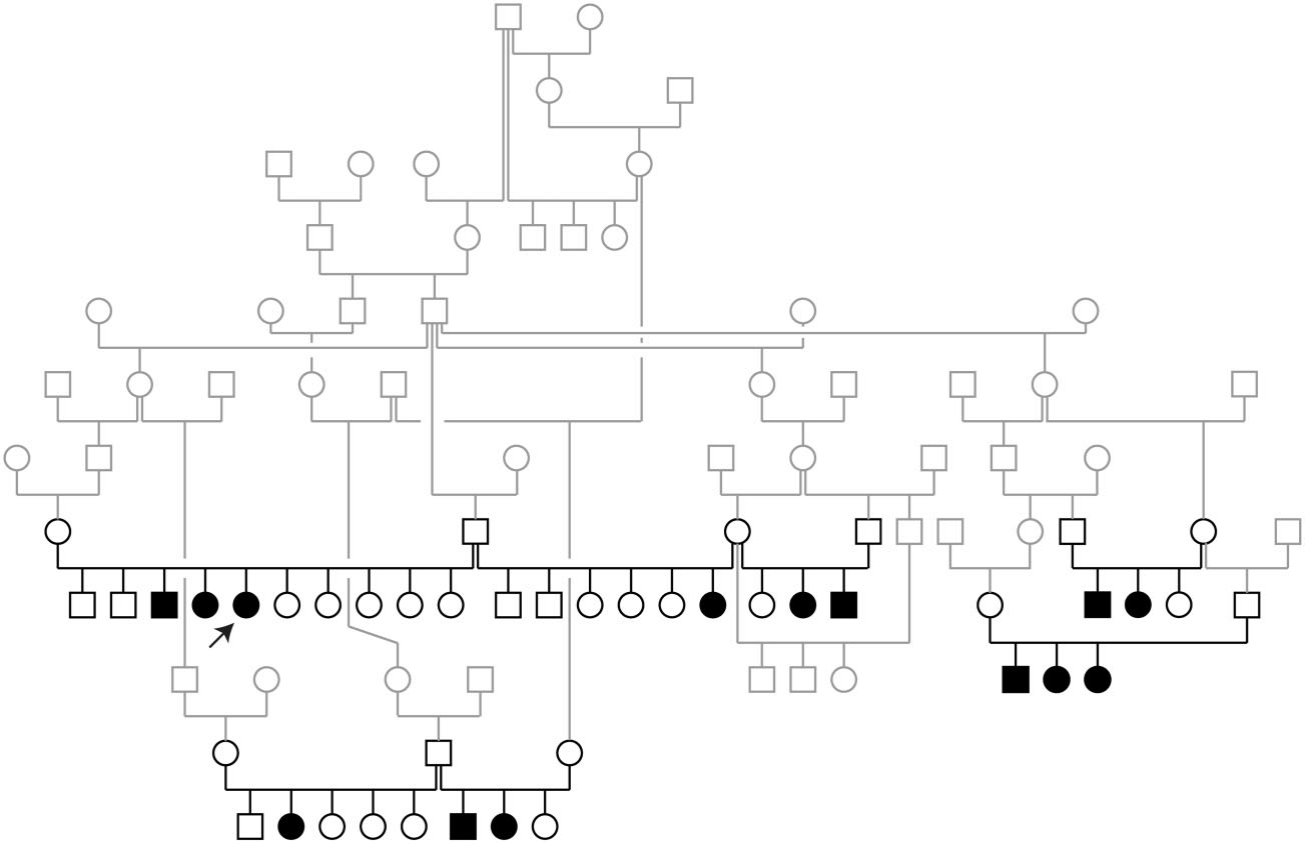
Pedigree of the investigated Golden Retriever family. Squares represent males and circles represent females. The affected dogs are indicated by filled symbols. Note the multiple inbreeding loops within this pedigree. All affected dogs have shared common ancestors in their maternal and paternal lineages. The 44 dogs that were genotyped on microarrays and used for the linkage analysis are indicated in black and constitute four separate subfamilies. The other dogs are shown in gray. The arrow indicates the dog that was used for whole-genome sequencing.

The 14 available cases belonged to seven different litters, each with unaffected parents. Both male and female dogs were affected. The pedigree showed multiple inbreeding loops and was strongly suggestive for a monogenic autosomal recessive inheritance (Figure 3).

**Figure 4:**
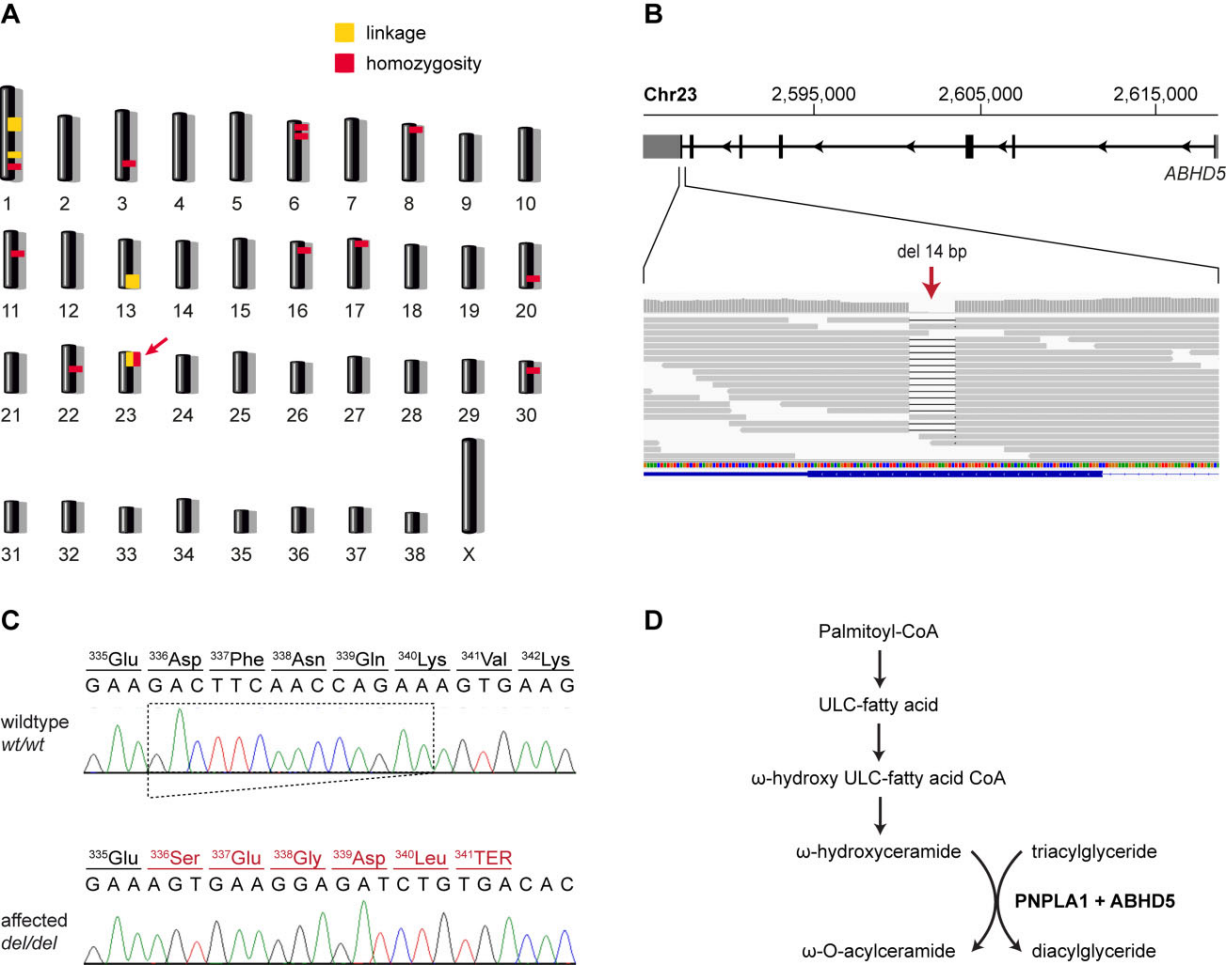
Mapping of the ICH2 locus and details of the *ABHD5*:c.1006_1019del variant. (A) Combined linkage and homozygosity mapping revealed a single overlapping region on chromosome 23 indicated by an arrow. (B) Integrative Genomics Viewer (IGV) screenshot showing the position of the deletion in the short-read alignments of the ICH2 affected dog. (C) Sanger sequencing confirmed the detected 14 bp frameshift deletion. The altered reading frame and the premature stop codon are indicated in red. (D) Metabolic pathway for the synthesis of ω-O-acylceramide, an essential lipid required to maintain skin barrier function (Haydar Eskiocak *et al.* 2019). In the last step of this pathway, ABHD5 acts as a coactivator of PNPLA1 and may help to provide the required triacylglycerides within the endoplasmic reticulum (Ohno *et al.* 2018). A lack of either ABHD5 or PNPLA1 leads to ichthyosis in humans (Lefevre *et al.* 2001; Grall *et al.* 2012).

By combining linkage analysis and homozygosity mapping, we determined the critical interval for the disease-causing genetic variant. A single ∼12.7 Mb segment on chromosome 23 showed both linkage in the families and shared homozygous genotypes in all 14 available cases (Figure 4A). The LOD score of the linked interval on chromosome 23 was 2.75 (Table 1, Supplementary Figure S1). The exact coordinates of the critical interval were Chr23:1–12,734,912 (Supplementary Table S1).

**Table 1:** Results of linkage analysis in 44 dogs from 7 litters and their parents

Chrom	Start (Mb)	Stop (Mb)	Max LOD score	α^*a*^
1	41.498	48.203	0.43	0.65
1	69.318	69.332	3.46	1
1	105.516	106.520	0.23	0.63
13	57.599	63.242	1.07	0.73
23	0.000	12.822	2.75	1

a α indicates an estimated proportion of the four subfamilies that show linkage. The dogs were organized in four subfamilies (see Materials and Methods and Figure 3).

The genome of one affected dog was sequenced at 26x coverage. Assuming that the disease allele is rare in the dog population, we filtered for private homozygous variants that were absent from 795 control genomes (Supplementary Table S2). This analysis identified 32 private homozygous variants in the critical interval, of which only one was predicted to be protein changing (Table 2, Supplementary Table S3).

**Table 2:** Results of variant filtering in the ICH2 affected Golden Retriever against 795 control genomes

Filtering step	Variants
All variants in the affected Golden Retriever	3,040,308
Private variants	1,110
Private variants in critical interval	32
Private protein-changing variants in critical interval	1

Only homozygous variants are reported.

The identified candidate variant was a 14 bp deletion in the last exon of *ABHD5*, XM_542689.5:c.1006_1019del (Figure 4, B and C). The formal genomic designation of the variant is Chr23:2,587,000–2,587,013del (CanFam3.1). It causes a frameshift, resulting in a premature stop codon and altering the last 14 codons of the open reading frame, XP_542689.2:p.(Asp336Serfs*6).

We confirmed the presence of this variant in the index case by Sanger sequencing and genotyped all 85 relatives. The genotypes at the variant showed perfect cosegregation with the ICH2 phenotype. All 14 available cases were homozygous for the deletion. The 11 parents of the 14 cases who represent obligate carriers were all heterozygous. The remaining 61 unaffected relatives were either heterozygous or homozygous for the wildtype allele. The mutant allele was absent from 396 Golden Retrievers of European origin (Table 3, Supplementary Table S4).

**Table 3:** Genotypes at the *ABHD5*: c.1006_1019del variant in Golden Retrievers of North American and European origin

Dogs	wt/wt	wt/del	del/del
ICH2 affected Golden Retrievers (*n* = 14)	—	—	14
Obligate carriers for ICH2 (*n* = 11)	—	11	—
Unaffected other relatives (*n* = 61)	35	26	—
Golden Retrievers of European origin (*n* = 396)	396	—	—

## Discussion

In this study, we investigated 14 Golden Retrievers with a new form of ichthyosis termed ICH2 that so far appears to be limited to dogs from North America. Histopathology classified ICH2 as nonepidermolytic ichthyosis. The 14 available cases came from seven different litters, all of which were related. The clinical and histological presentation of ICH2-affected dogs strongly resembled that of the well-known *PNPLA1*-related ichthyosis in Golden Retrievers (Grall *et al.* 2012). Experienced breeders reported more severe and adherent scaling in ICH2-affected Golden Retrievers. Histologically, the epidermis in ICH2-affected dogs is thicker than in dogs with the *PNPLA1*-related ichthyosis dogs and this may correspond with a more severe barrier defect. ICH2 cases tend to have more keratinocytes with perinuclear clear spaces than dogs with the *PNPLA1*-related ichthyosis.

Using a hypothesis-free positional approach we delimited a ∼12.7 Mb interval or roughly 0.5% of the 2.4 Gb dog genome for the ICH2 locus. Contrasting the genome sequence of an affected dog against 795 control genomes identified a single private homozygous coding variant within the critical interval, a 14 bp deletion in the last exon of the *ABHD5* gene. *ABHD5* encodes α-β hydrolase domain containing 5 also known under the alias name CGI-58. This protein is involved in the biosynthesis of major components of the skin barrier formation, especially ω-O-acylceramide (Ghosh *et al.* 2008; Ohno *et al.* 2018). Studies in *Abhd5^-/-^* knockout mice showed drastically reduced triglyceride (TG) hydrolase activity in the epidermis. The defective epidermal TG catabolism led to an impaired synthesis of acylceramides and defective formation of the corneocyte lipid envelope resulting in a dysfunctional permeability barrier of the skin (Radner *et al.* 2010). ABHD5 also activates adipose triglyceride lipase (ATGL), which catalyzes the initial step of lipolysis converting TG to diglycerides (DG) (Zimmermann *et al.* 2004; Lass *et al.* 2006).

*ABHD5* loss-of-function variants in humans were reported to cause Chanarin-Dorfman syndrome (CDS) (OMIM # 275630, Dorfman *et al.* 1974; Chanarin *et al.* 1975; Lefevre *et al.* 2001). CDS is a rare autosomal recessive inherited syndromic form of ichthyosis. In CDS, a nonepidermolytic ichthyosis is often accompanied by hepatic steatosis with hepatomegaly, myopathy, and neurological disorders due to lipid droplet accumulation in various tissues (Dorfman *et al.* 1974; Chanarin *et al.* 1975; Bruno *et al.* 2008).

More than ten pathogenic human *ABHD5* variants have been described, many of which are affecting the α-β hydrolase domain, lipid binding region, pseudocatalytic domain, or acyltransferase domain (Lefevre *et al*. 2001; Schweiger *et al.* 2009). The *ABHD5*:c.1006_1019del frameshift deletion identified in the affected Golden Retrievers of this study alters the last 14 codons of the open reading frame. They include the last 10 codons of the α-β hydrolase domain (Schweiger *et al.* 2009). A comparable *ABHD5* nonsense variant truncating the last 14 codons of the homologous human sequence was reported in four CDS patients from a consanguineous family (Aggarwal *et al.* 2012). This strongly suggests an essential function of the C-terminal tail of ABHD5 and supports the hypothesis that the observed *ABHD5*:c.1006_1019del frameshift deletion is indeed causative for the ichthyosis in the investigated Golden Retrievers.

In summary, we discovered a new autosomal recessive ichthyosis in Golden Retrievers, which we propose to designate as Golden Retriever ichthyosis type 2 (ICH2). The identified candidate causative variant enables genetic testing to prevent the unintentional breeding of affected puppies. Further studies are warranted to clarify whether the phenotypic changes in the affected dogs are limited to the skin or whether ICH2 also affects other organs like CDS in humans.

## Data availability

The genome sequence data were submitted to the European Nucleotide Archive (ENA). All accession numbers are listed in Supplementary Table S2.

Supplementary material is available at *G3* online.

## Supplementary Material

jkab397_Supplementary_Data
