# Supplementary material for: ABHD5 frameshift deletion in Golden Retrievers with ichthyosis
Source: G3 (Bethesda). 2021 Nov 15;12(2):jkab397. doi: 10.1093/g3journal/jkab397 (PMC9210301; doi:10.1093/g3journal/jkab397)
Supplement: jkab397_Supplementary_Data [file jkab397_supplementary_data.zip › GENETICS-G3-2021-402919-s03.pdf]

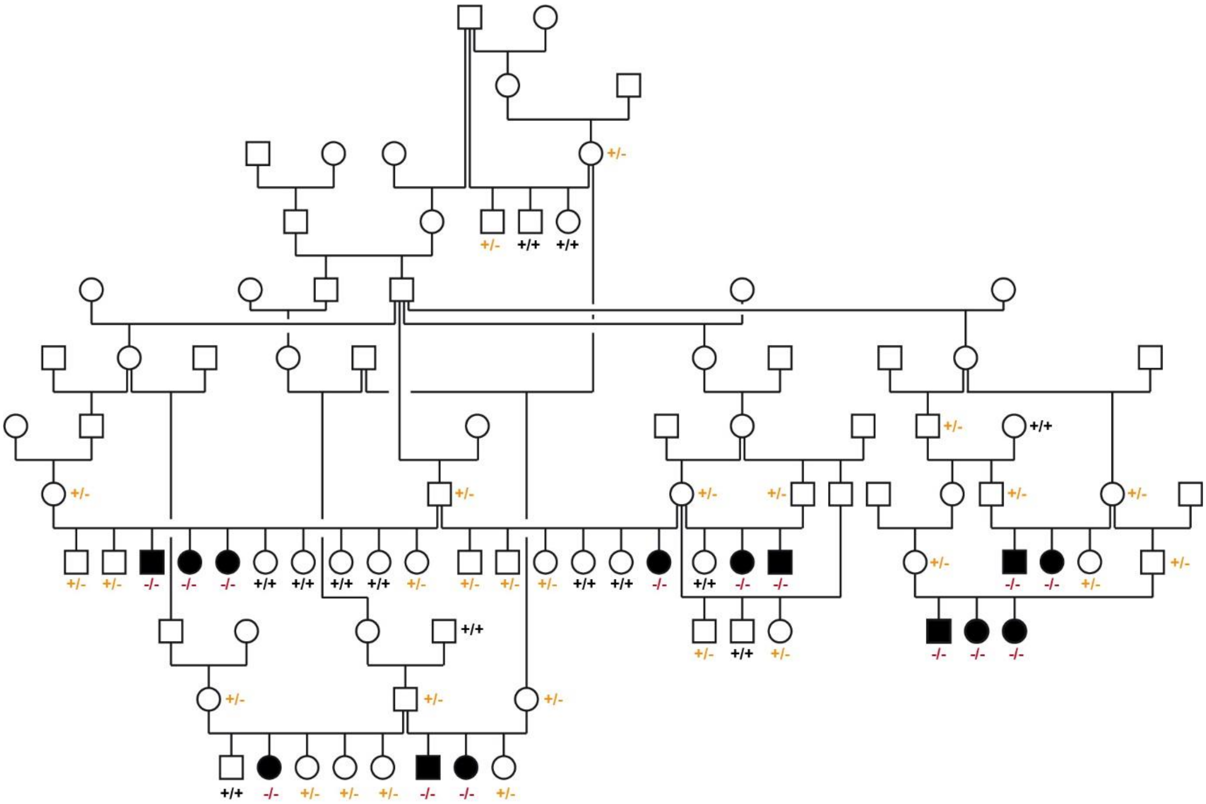

Figure S2. Pedigree with *ABHD5*:c.1006_1019del genotypes from 54 dogs. +/+ denotes homozygous wildtype, +/- is heterozygous and -/- represents homozygous mutant genotypes. For this study, 86 related dogs were genotyped. A total of 32 unaffected dogs and their genotypes are not represented in this pedigree as it would otherwise have become too large to fit on one page. Note the perfect co-segregation of the genotypes with the ichthyosis phenotype. All 14 affected dogs carried the deletion in a homozygous state. The unaffected dogs were all either homozygous wildtype or heterozygous. A total of 11 obligate carriers comprising all parents of the 7 available litters with affected puppies were all heterozygous.
