# Supplementary material for: ABHD5 frameshift deletion in Golden Retrievers with ichthyosis
Source: G3 (Bethesda). 2021 Nov 15;12(2):jkab397. doi: 10.1093/g3journal/jkab397 (PMC9210301; doi:10.1093/g3journal/jkab397)
Supplement: jkab397_Supplementary_Data [file jkab397_supplementary_data.zip › GENETICS-G3-2021-402919-s02.pdf]

Figure S1. Linkage analysis.

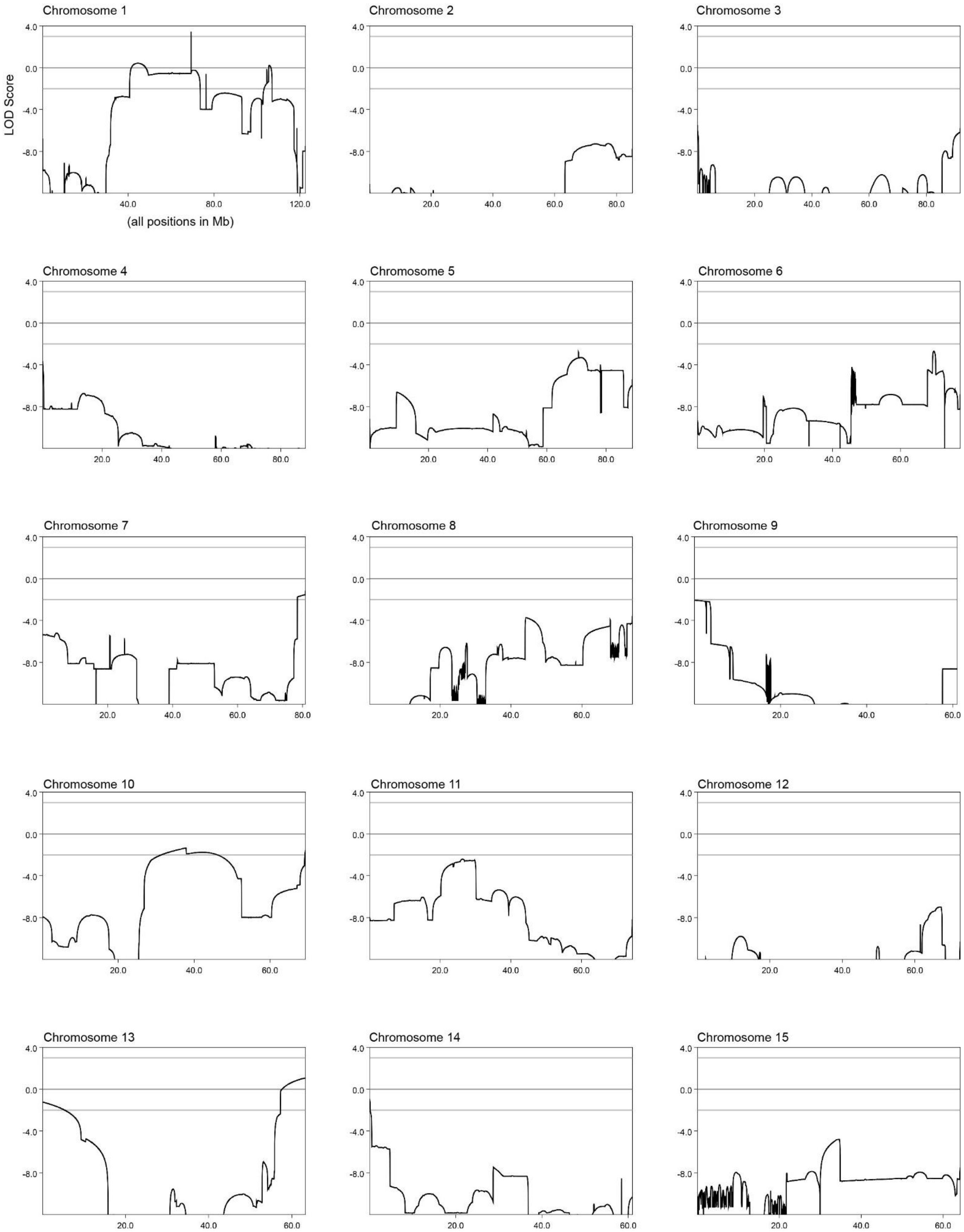

Figure S1. Linkage analysis.

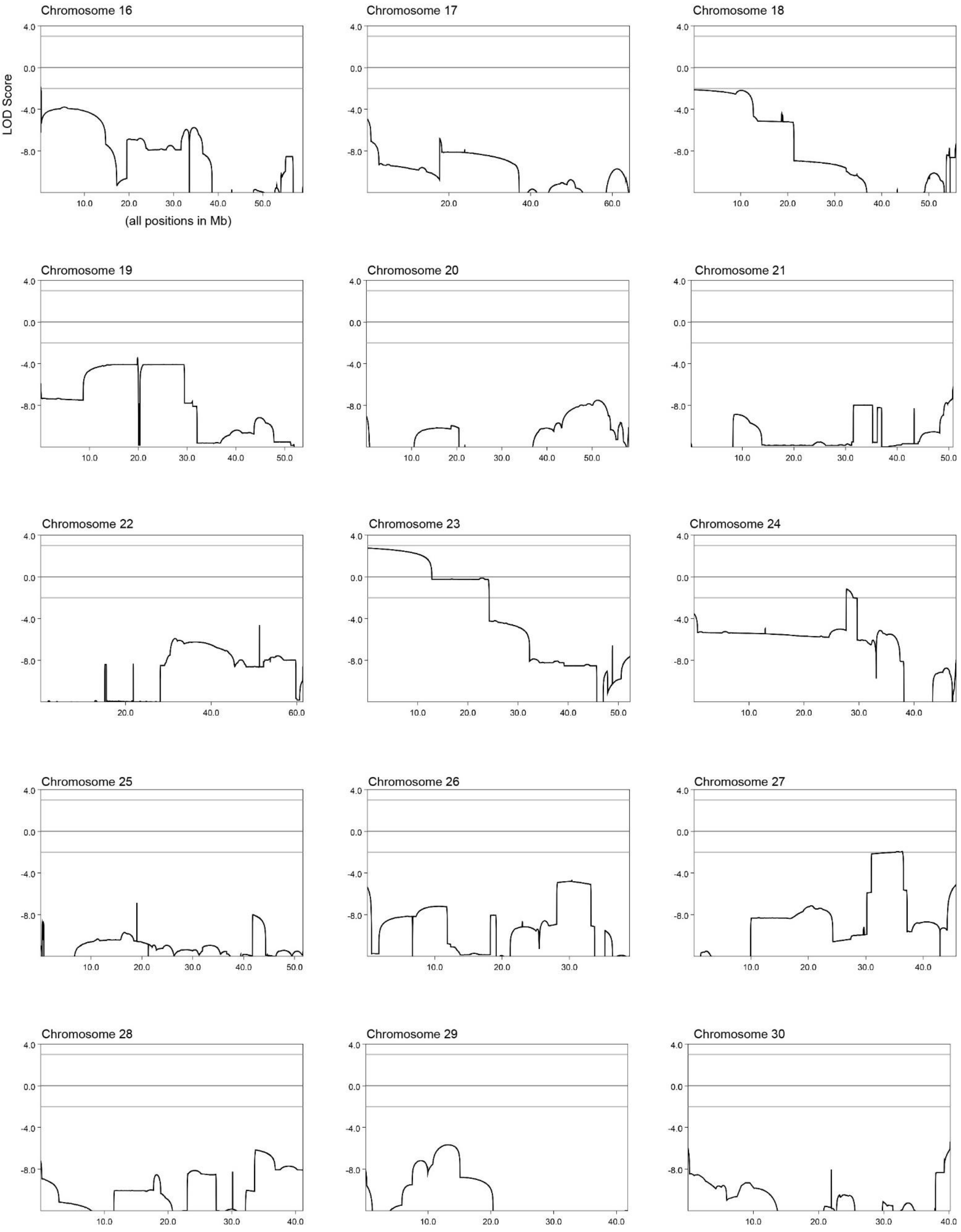

Figure S1. Linkage analysis.

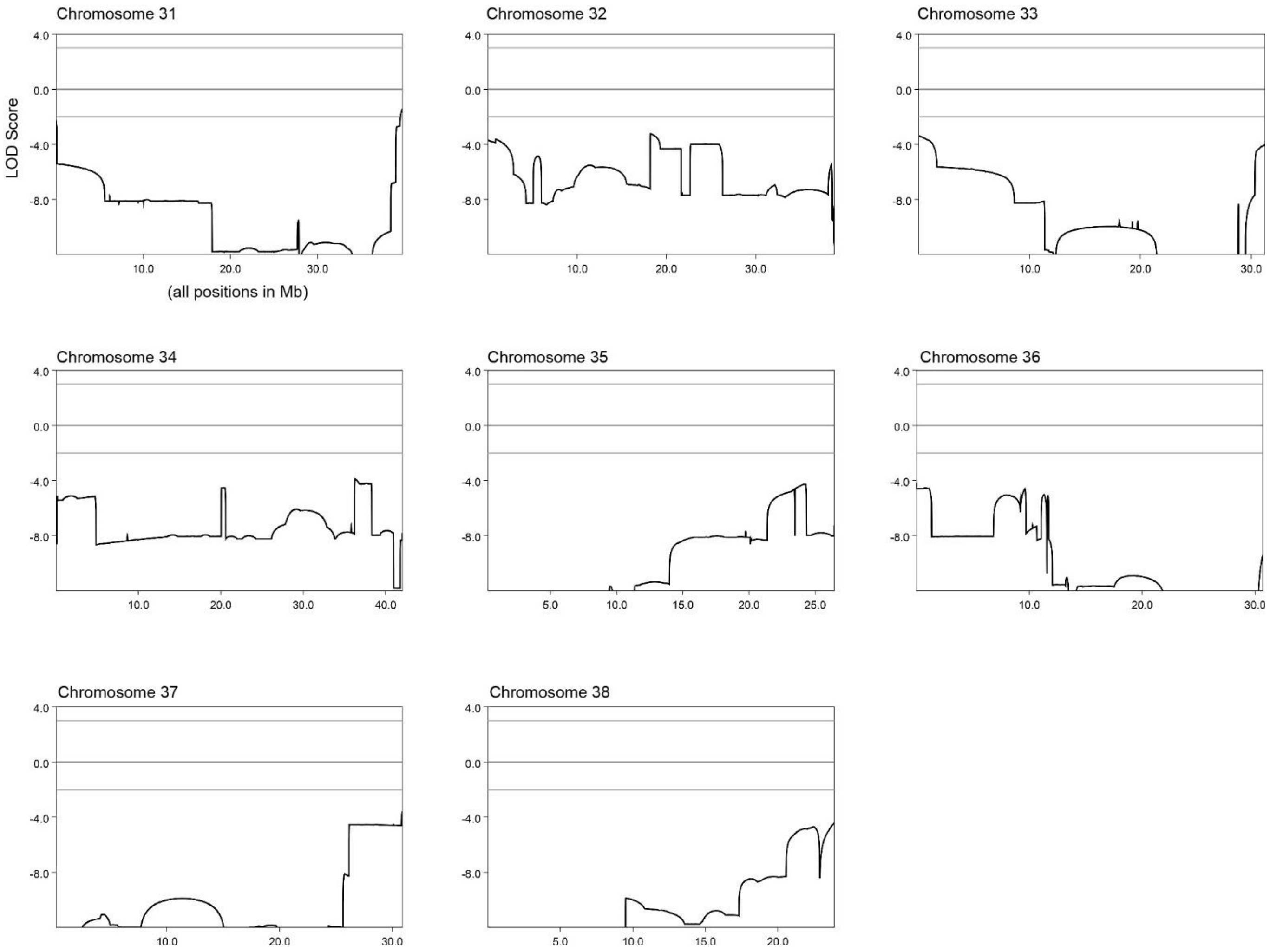

Figure S1. Linkage analysis. A total of 44 animals (dogs from 7 litters comprising 11 unaffected parents, 14 affected and 19 unaffected offspring) were used for the analysis. Parametric linkage analysis indicated two linked regions: A very small region on chromosome 1 (~14 kb, 3 markers) and a ~12.8 Mb region on chromosome 23. Only the linked region on chromosome 23 coincided with an extended interval of shared homozygous haplotypes in the cases. All positions correspond to the CanFam 3.1 reference genome assembly.
